# High Prevalence of Intermediate *Leptospira* spp. DNA in Febrile Humans from Urban and Rural Ecuador

**DOI:** 10.3201/eid2112.140659

**Published:** 2015-12

**Authors:** Jorge Chiriboga, Verónica Barragan, Gabriela Arroyo, Andrea Sosa, Dawn N. Birdsell, Karool España, Ana Mora, Emilia Espín, María Eugenia Mejía, Melba Morales, Carmina Pinargote, Manuel Gonzalez, Rudy Hartskeerl, Paul Keim, Gustavo Bretas, Joseph N.S. Eisenberg, Gabriel Trueba

**Affiliations:** Microbiology Institute, Universidad San Francisco de Quito, Campus Cumbaya, Quito, Ecuador (J. Chiriboga, V. Barragan, G. Arroyo, A. Sosa, E. Espín, M.E. Mejía, G. Trueba);; Northern Arizona University, Flagstaff, Arizona, USA (D.N. Birdsell, P. Keim);; Instituto Nacional de Salud Pública e Investigación, Portoviejo, Ecuador (K. España, A. Mora, M. Morales, C. Pinargote, M. Gonzalez)**;**; Ministerio de Salud Pública, Portoviejo (M. Morales);; Royal Tropical Institute (KIT), Amsterdam, the Netherlands (R. Hartskeerl)**;**; Organización Panamericana de la Salud OPS, Guayaquil, Ecuador (G. Bretas);; University of Michigan, Ann Arbor, Michigan, USA (J.N.S. Eisenberg)

**Keywords:** Leptospira spp., Ecuador, intermediate cluster, intermediate species, fever, carrier animals, rrs gene, urban areas, rural areas, semiurban areas, bacteria, humans, febrile, zoonoses, prevalence

## Abstract

*Leptospira* spp., which comprise 3 clusters (pathogenic, saprophytic, and intermediate) that vary in pathogenicity, infect >1 million persons worldwide each year. The disease burden of the intermediate leptospires is unclear. To increase knowledge of this cluster, we used new molecular approaches to characterize *Leptospira* spp. in 464 samples from febrile patients in rural, semiurban, and urban communities in Ecuador; in 20 samples from nonfebrile persons in the rural community; and in 206 samples from animals in the semiurban community. We observed a higher percentage of leptospiral DNA–positive samples from febrile persons in rural (64%) versus urban (21%) and semiurban (25%) communities; no leptospires were detected in nonfebrile persons. The percentage of intermediate cluster strains in humans (96%) was higher than that of pathogenic cluster strains (4%); strains in animal samples belonged to intermediate (49%) and pathogenic (51%) clusters. Intermediate cluster strains may be causing a substantial amount of fever in coastal Ecuador.

## Abstract 2

Intermediate clusters may cause disease in areas where dengue and malaria are present, so differential diagnosis is necessary.

Leptospirosis, caused by spirochetes of the genus *Leptospira*, is a neglected and potentially fatal disease that burdens impoverished communities of developing nations in tropical regions (*1*–*4*). The bacteria cause 1.7 million human cases of severe disease worldwide each year (*1*,*2*); outbreaks frequently occur during the rainy season in cities in the tropics (*4*–*8*). Domestic, peridomestic, and wild mammals harbor diverse *Leptospira* spp. in their kidneys, and their urine contaminates water sources and soil (*6*,*8*).

*Leptospira* comprises 20 species that are phylogenetically arranged in 3 clusters: pathogenic, saprophytic, and intermediate (*6*,*9*). Nine pathogenic and 5 intermediate species, comprising >200 serovars, have been characterized (*6*,*9*,*10*). Some reports associate intermediate cluster strains with mild (*11*–*14*) to severe (*15*,*16*) leptospirosis; however, this cluster is not well characterized (*3*,*11*,*15*,*16*). Furthermore, the current notion is that human leptospirosis is mainly caused by strains of the pathogenic cluster (*2*,*4*,*6*,*9*,*10*).

Many aspects of leptospirosis epidemiology remain unknown because only limited information exists regarding leptospiral population genetics and the role of environmental factors, including environmental persistence of leptospires, in disease occurrence. These deficiencies in knowledge result from the complexity of the disease (e.g., many animal reservoirs carry 1 of the 14 species of potentially infectious leptospires) and technical difficulties associated with classical diagnostics, such as cumbersome isolation of bacteria from clinical samples, complex standard serologic methods, and a lack of culture techniques to obtain isolates from environmental samples. We present a molecular approach to address some of these shortcomings.

Leptospirosis is common in tropical areas of Ecuador (*17*). The most severe documented outbreak occurred in 1998 in Guayaquil, where 80% of case-patients required hospitalization and 12% died (J. Leake, pers. comm., 2004). During 2010–2012 in Portoviejo, Ecuador, >2,000 serologically confirmed cases of febrile leptospirosis were reported by local health authorities (M. Morales, pers. comm., 2013). We used molecular methods to amplify and sequence the leptospiral 16S *rrs* gene from clinical samples from patients in 3 coastal communities in Ecuador that vary in their levels of urbanization.

## Methods

### Human Samples

During February 2011–December 2012, a total of 464 serum and blood spot samples were collected from acute, febrile patients attending hospitals or health posts in rural, semiurban, and urban communities in Ecuador. Samples from Esmeraldas, a rural community, were provided by Hospital de Borbón (Esmeraldas Province) and the Ecuador Ministry of Health (MoH). The hospital provided 108 serum samples from febrile patients; the samples had been tested for dengue virus (IgM ELISA; PanBio, Brisbane, Queensland, Australia) but not *Leptospira* spp.; 33 were positive for dengue virus. During the same time period, the Ecuador MoH collected 102 blood spot samples from febrile patients in Esmeraldas. The samples were collected onto filter paper (Whatman 903 Specimen Collection Paper; Whatman, Springfield Mill, UK), dried at room temperature, and stored at −20°C in plastic zipper bags. Twenty serum samples obtained from nonfebrile persons during March 2012 (rainy season) in the same locality were also provided. Protocols used to obtain human samples from Esmeraldas were approved by the Universidad San Francisco de Quito Bioethics Committee and the University of Michigan Institutional Review Board.

A total of 100 serum samples from febrile patients in Portoviejo, a semiurban community, were provided by the Ecuador MoH; 34 were positive for *Leptospira* spp. (IgM ELISA; PanBio). The other 66 samples were not tested for *Leptospira* spp., but they were tested for dengue virus by IgM ELISA (9 were positive). The samples had been collected during the rainy season, March–June 2012.

A total of 154 serum samples from febrile patients in Guayaquil, an urban community, were provided by the Ecuador MoH. Samples were collected from different medical posts and hospitals around the city during the rainy season, July–October 2011. The samples had been tested for dengue virus by IgM ELISA (all were negative); no samples were tested for *Leptospira* spp. All samples from Portoviejo and Guayaquil were collected by government officials and were deidentified before being sent to our laboratory.

### Animal Samples

In Portoviejo, during the dry season in 2009 and the wet season in 2013, we collected urine samples from domestic animals (27 pigs, 30 dogs, and 27 cows in 2009; 30 pigs and 26 cows in 2013) and kidney samples from rats (6 in 2009 and 60 in 2013). We administered 2.5 mg/kg of furosemide (a diuretic) to animals to collect their urine during micturition or by cystocentesis. Rats were captured inside the homes of Portoviejo residents by using traps from Tomahawk Live Trap (Hazelhurst, WI, USA) or snap traps, and as needed, they were euthanized by using chloroform. Urine samples collected in 2009 from cattle, pigs, and dogs were obtained from residential areas. Cattle and pig urine samples collected in 2013 were obtained from a local slaughterhouse that processed animals from the same location.

### Overview of the Molecular Analyses

Our overall analytic goal was to ensure detection of leptospires of the intermediate and pathogenic clusters. To this end, we adapted a previously used protocol to amplify the *rrs* genes from pathogenic and intermediate clusters. We then sequenced the *rrs* gene to detect leptospiral species and anomalous amplification products. A sample was considered positive when its amplicon comprised sequences for leptospira bacteria.

### DNA Extraction

Frozen animal urine samples (10 mL) were thawed on ice and pelleted by centrifugation at 3,287 × *g* for 15 min. DNA was extracted from the pellets by using the QIAamp DNA Mini Kit (QIAGEN, Valencia, CA, USA) and stored at −80°C. Frozen serum samples were thawed on ice, and 200 μL was used for DNA extraction (QIAamp DNA Mini Kit); the DNA was stored at −80°C. Eight punches (6-mm diameter) from blood spots were placed in a 1.5-mL microcentrifuge tube and incubated in 180 μL of ATL buffer (QIAGEN) for 10 min at 85°C, and the supernatant was transferred into a new 1.5-mL microcentrifuge tube and processed for DNA extraction.

A 2-mm^3^ section of rat kidney was cut and washed 3 times with 1 mL of PBS. DNA was extracted by dissolving the kidney tissue in 700 µL of CTAB extraction buffer, followed by incubation (with shaking every 15 min) for 2 h at 65°C. The tubes were cooled to room temperature, and 700 µL of a chloroform–isoamyl alcohol (24:1) mixture was added to each tube. Contents were mixed and then centrifuged at 6,000 × *g* for 5 min, and the aqueous phase was transferred to another tube. DNA was precipitated with a 3 M sodium acetate (pH 5) solution and ethanol, and the pellet was washed with 70% ethanol, dried, and dissolved in 50 μL of Tris-EDTA buffer.

### Amplification of Leptospiral *rrs* Gene

Leptospiral DNA from samples was detected by using 1 of the following primer sets (AB or CD), both of which amplify the same small fragment target of 16S *rrs* gene specific to leptospiral species: forward A 5′-GGCGGCGCGTCTITAAACATG-3′, reverse B 5′-TTCCCCCCATTGAGCAAGATT-3′, forward C 5′-CAAGTCAAGCGGAGTAGCAA-3′, reverse D 5′-CTTAACCTGCTGCCTCCCGTA-3′ (*18*). Amplicon sizes were 332 bp for primers AB and 290 bp for primers CD. We adapted this protocol for real-time PCR using the CFX96 Real-Time PCR Detection System (Bio-Rad, Hercules, CA, USA). The PCR master mix included iQ SYBR Green Supermix (Bio-Rad), 0.5 μM each primer and molecular biology–grade water, and 1 μL of DNA template for a final reaction volume of 10 μL. Our amplification protocol used an initial enzyme activation step at 95°C for 3 min, followed by 45 amplification cycles (30 s at 95°C, 30 s at 62.5°C, 30 s at 72°C). To detect the presence of amplified *rrs* gene amplicon, we performed a melting curve analysis (65°C to 85°C, with a ramp of 0.5°C/5 s). To increase the concentration of the *rrs* gene amplicon, we subjected the PCR products from samples positive for *rrs* gene amplification to a second round of PCR amplification by using the conventional PCR protocol (*18*). Using the same AB or CD primer pairs and 0.5 μL of *rrs* gene amplicons from the first PCR amplification, we initiated the amplification protocol for the second PCR with denaturation at 94°C for 3 min, followed by 29 cycles of 94°C for 1 min, 63°C for 1.5 min, and 72°C 2 min, and then ended with a final 10-min elongation at 72°C. To rule out accidental contamination of PCR reagents, we included negative controls in all reactions. In addition, all reagents used in our analyses performed during 2012–2013 were subjected DNA amplification by using primer sets AB and CD (real-time and conventional PCR).

### Sequence Analysis of Leptospiral *rrs* Gene

Concentrated *rrs* gene amplicons of 277 samples were sequenced at Functional Biosciences (Madison, WI, USA) by using primers AB or CD. To analyze DNA sequences, we used MEGA 5.08 (http://www.megasoftware.net) and BLAST (http://blast.ncbi.nlm.nih.gov/Blast.cgi). For sequencing, we selected 19 amplicons from samples collected in 2009 from different animals in Portoviejo. Overall, 11 *Leptospira* spp. sequences were submitted to GenBank under accession nos. JN377490 and JN377491 (*L. inadai* from animals in Portoviejo, 2009); JN377492 (*L. borgpetersenii* from an animal in Portoviejo, 2009); KF303505 (*L. wolffii* from a human in Esmeraldas, 2012); KF285460 (*L. wolffii* from a human in Portoviejo, 2012); KF285460 (*L. wolffii* from a human in Guayaquil, 2011); KM259910 (*L. wolffii* from an animal in Portoviejo, 2013); KF303504 (*L. noguchii* from a human in Esmeraldas, 2012); KF303503 (*L. borgpetersenii* from a human in Guayaquil, 2011); KJ573104 (*L. noguchii* from an animal in Portoviejo, 2013); and KJ573105 (*L. borgpetersenii* from an animal in Portoviejo, 2013).

### Design of Intermediate *Leptospira* spp.–Specific Assay

We designed a *Leptospira* spp. assay to target only intermediate *Leptospira* spp. We used Primer3 (*19*) to design a reverse primer (R Inter: 5′-TCTTTACCTATCARATCYTGTGATCCA-3′) to be used with A or C forward primers; amplicon sizes were 160 bp for A and 143 bp for C. The specificity of this assay was validated with 17 leptospiral DNA samples from reference strains of pathogenic, saprophytic, and intermediate leptospiral species obtained from the Royal Tropical Institute (Table 1). We tested for the intermediate leptospiral genotype in 75 human serum samples from Portoviejo that were real-time PCR positive for *Leptospira* spp. The real-time PCR amplicons were subjected to a second PCR amplification using R-Inter reverse primer. The total reaction volume of the intermediate-specific real-time PCR assay, using GoTaq Flexi Polymerase (Promega, Madison, WI, USA), was 20 μL; 0.5 μL of the real-time PCR amplicon was used as template for the conventional PCR.

**Table 1:** PCR results by using primer Inter- R combined with primers A and C*

*Leptospira *spp.	Strain	PCR result	
Intermediate		
* L. broomii*	5399	+
* L. fainei*	BUT 6	+
* L.inadai*	10	+
* L. wolffii*	Korat-H2T	+
* L. licerasiae*	VAR010	+
Pathogenic		
* L. interrogans*	Pomona	−
* L. kirschneri*	Kambale	−
* L. borgpetersenii*	MUS 127	−
* L. noguchii*	M7	−
* L. alexanderi*	A85	−
* L. santarosai*	CZ 390	−
* L. weilii*	Sarmin	−
Saprophytic		
* L. vanthielii*	WazHolland	−
* L. biflexa*	Patoc I	−
* L.meyeri*	ICF	−
* L. wolbachii*	CDC	−
* L. kmetyi*	Bejo-Iso9T	−

*Only intermediate *Leptospira* species were amplified by using Inter-R specific primer. +, positive; –, negative.

## Results

### Human Samples

Leptospiral DNA was detected in 73 (68%) of 108 serum samples and 59 (57%) of 102 blood spots from febrile patients in the rural study site (Esmeraldas) (Table 2; Figure). All *Leptospira* spp.–positive amplicons from blood spots and 70 (96%) of the 73 *Leptospira* spp.–positive amplicons from serum samples showed 100% DNA sequence identity with *L. wolffii* (intermediate cluster). The remaining 3 positive amplicons from serum samples showed 99% identity with *L. noguchii* (pathogenic cluster). Of the 108 serum samples, 31 (29%) were positive for *Leptospira* spp. (PCR) and dengue virus (IgM ELISA), 4 (3.7%) were positive for dengue virus only (IgM ELISA), and 42 (39%) were positive for *Leptospira* spp. only (PCR). DNA sequences of 6 (4%) of 135 amplicons showed anomalous amplification products. All serum samples from nonfebrile patients were either PCR negative for *Leptospira rrs* gene (n = 15) or determined to be negative for *Leptospira* spp. because of anomalous amplification products (n = 5).

**Table 2:** *Leptospira* spp.–positive samples from febrile patients in 3 communities along the coast of Ecuador, 2011–2012*

Location, year	No. samples analyzed	*Leptospira* spp.–positive samples					No. (%) spurious PCR products†
		Pathogenic cluster			Intermediate cluster		
		No. (%)	Species		No. (%)	Species	
Esmeraldas, 2011–2012‡	108§	3 (2.7)	*L. noguchii*		73 (68)	*L. wolffii*	6 (4)
	102¶	0	–		59 (58)	*L. wolffii*	0
Portoviejo, 2012#	100	0	–		24 (24)	*L. wolffii*	0
					1 (1)	*L. inadai*	15 (32)
Guayaquil 2011**	154	3 (1.9)	*L. borgpetersenii*		28 (18)	*L. wolffii*	9 (21)
		1 (0.6)	*L. kirschneri*/*L. interrogans*††		–	–	0

*The 3 communities were in rural (Esmeraldas), semiurban (Portoviejo), and urban (Guayaquil) locations. Leptospiral DNA in patient samples was detected by PCR. Molecular methods were used to amplify and sequence the leptospiral *rrs* gene from DNA. –, not applicable/no value. †The spurious products represent serum samples that produced amplicons of the correct size but with DNA sequences different from *Leptospira* (for the pathogenic and intermediate cluster). ‡Of samples from Esmeraldas, 27% were positive for dengue virus (IgM ELISA) and *Leptospira* sp. (PCR). §Serum samples.  ¶Blood spot samples. #Sixty-six samples were tested for dengue virus by IgM ELISA (57 negative, 9 positive) but were not tested for *Leptospira* sp.; 34 samples were IgM ELISA–positive for *Leptospira* sp. but were not tested for dengue virus. **Samples tested negative for dengue virus by IgM ELISA. ††The amplicon showed the same degree of identity to both species.

**Figure:**
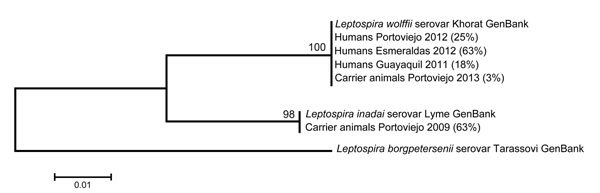
Maximum-likelihood tree for DNA sequences of the *Leptospira* spp. *rrs* gene recovered from serum samples from febrile humans and from urine and kidney samples from animal carriers in Ecuador. Esmeraldas, Portoviejo, and Guayaquil are 3 rural, semiurban, and urban communities, respectively, along the coast of Ecuador. Pathogenic *L. borgpetersenii* was used as an outgroup. Numbers in parentheses indicate the percentage of samples per community that contained DNA signatures highly similar to GenBank reference strains *L. wolffii* (NR_044042), *L. inadai* (accession no. JQ988844.1), and *L. borgpetersenii* (accession no. JQ988861.1). Scale bar indicates the degree of nucleotide substitutions.

Leptospiral DNA was detected in 25 (25%) of 100 serum samples from the semiurban study site (Portoviejo): 7 of these were also IgM positive for *Leptospira* spp. (dengue IgM unknown), 16 were IgM ELISA negative for dengue virus (*Leptospira* spp. IgM unknown), and 2 were IgM ELISA positive for dengue virus (*Leptospira* IgM unknown). Twenty-four *Leptospira* spp.–positive amplicons showed 100% DNA sequence identity to *L. wolffii*, 1 amplicon showed 98% identity to *L. inadai*, and 15 amplicons (of the expected size) were anomalous amplification products.

Leptospiral DNA was detected in 32 (21%) of 154 serum samples from the urban study site (Guayaquil) (Table 2). As with samples from Portoviejo and Esmeraldas, most samples from Guayaquil had amplicon sequences that shared 100% identity with *L. wolffii* (intermediate cluster). Only 3 (2%) samples shared amplicon sequence identity with pathogenic *Leptospira* spp. and 2 with *L. borgpetersenii* (99% identity); 1 could not be differentiated as *L. kirschneri* or *L. interrogans* (both 99% identity) (Table 2). Of 43 amplicons displaying the expected size, 9 were anomalous amplification products.

### Animal Samples

Of the 90 animal samples collected from Portoviejo during the 2009 dry season, 65 (72%) were PCR positive for *Leptospira* spp.: 21 (70%) of 30 samples from dogs, 18 (67%) of 27 from pigs, 20 (74%) of 27 from cattle, and all 6 rat kidney samples. However, we sequenced only 19 amplicons from these samples (3 from dogs, 3 from pigs, 7 from cattle, and 6 from rats). BLAST analysis of amplicon sequences from these 19 samples showed that 14 (74%) had 100% sequence identity to *L. inadai* (intermediate cluster), whereas amplicons from 5 animals (3 cows, 1 pig, and 1 rat) had 100% identity to *L. borgpetersenii* (pathogenic cluster) (Figure; Table 3). During 2009–2013, the dominant species of leptospires shifted from *L. inadai* (intermediate cluster) to *L. borgpetersenii* (pathogenic cluster) (Table 3). In addition, among intermediate types, we observed a population shift from *L. inadai* to *L. wolffii* (Table 3; Figure); the identity for *L. wolffii* sequences was 99%. 

**Table 3:** Species and cluster of leptospiral DNA sequences recovered from animals in 2009 and 2013, Portoviejo, Ecuador*

Location, year, animal, no analyzed samples	*Leptospira* spp.–positive samples					No. (%) spurious PCR products†
	Pathogenic cluster			Intermediate cluster		
	No. (%)	Species		No. (%)	Species	
Portoviejo, 2009‡						
Cattle, n = 7	3 (43)	*L. borgpetersenii*		4 (57)	*L. inadai*	0
Rats, n = 6	1 (17)	*L. borgpetersenii*		5 (83)	*L. inadai*	0
Dogs, n = 3	0	–		3 (100)	*L. inadai*	0
Pigs, n = 3	1 (33)	*L. borgpetersenii*		2 (67)	*L. inadai*	0
Portoviejo, 2013§						
Cattle, n = 26	5 (19)	*L. borgpetersenii*		1 (4)	*L.wolffii*	3 (27)
	1 (4)	*L. kirschneri*		–	–	
Rats, n = 60	3 (5)	*L. borgpeterseni *		1 (1.7)	*L.wolffii*	3 (21)
	2 (3.3)	*L. kirschner¶*		–	–	
Pigs, n = 30	2 (6.7)	*L. borgpetersenii*		1 (3.3)	*L.wolffii*	5 (50)
*–, not applicable/no value. †Percentage of amplicons (obtained from samples of each animal species) which showed expected size but the DNA sequences were different from *Leptospira* spp. ‡Dry season. §Rainy season. ¶One amplicon also showed the same degree of identity to *L. interrogans*.						

### Verification of the Intermediate *Leptospira* spp.*–*Specific Assay

The R-Inter primers amplified only intermediate *Leptospira* sequences when tested against 17 leptospiral DNA from reference strains (Table 1). Of the 75 human serum samples with a supportive real-time PCR melting curve, 12 were positive for leptospiral sequence when primer pair AB was used, but 23 were positive when primer R-Inter was used. Of the 12 PCR reaction products that were positive by primer pair AB, 10 were also positive when using primer pairs A/R-Inter or C/R-Inter, and 2 DNA samples positive for leptospiral sequence using primers AB were negative when using R-Inter primer. The amplified sequences showed 100% identity to *L. wolffii.* We did not run this test with *L. inadai*–positive samples collected from animals in Portoviejo in 2009 because the samples were unavailable for this analysis. Nevertheless, in silico testing showed that the nucleotide sequence of R-Inter primer was identical to *L. inadai* sequences.

## Discussion

Our findings show that leptospiral DNA was present in various proportions in febrile patients living in 3 communities in Ecuador; the DNA was present in 63% of samples from persons at a rural site and in 25% and 21% of samples from persons at semiurban and urban sites, respectively. The use of leptospiral *rrs* DNA amplification and subsequent sequencing enabled us to detect leptospiral DNA (pathogenic and intermediate clusters) and rule out false-positive reactions. Of note, 96% of leptospiral DNA from human serum showed identity with intermediate rather than pathogenic clade strains. This finding is in contrast with the current notion that human leptospirosis is mainly caused by pathogenic cluster strains (*2*,*4*,*6*,*9*,*10*). One reason our findings contrast with those of prior studies is that we sequenced the amplified *rrs* gene to identify false-positive reactions and to identify intermediate cluster *Leptospira* spp.

Although our study lacked serologic data to determine acute leptospirosis (seroconversion using paired serum samples), the presence of leptospiral DNA in febrile persons combined with no evidence of dengue infection (a major cause of fever in coastal Ecuador) makes it plausible that the fever was caused by leptospirosis. The finding that none of the serum samples from asymptomatic persons contained leptospiral DNA also supports this finding.

The presence of intermediate leptospiral DNA and the absence of more serious symptoms of leptospirosis (jaundice, hemorrhages, renal failure) in our study are consistent with reports of mild disease linked to intermediate *Leptospira* spp., such as *L. licerasiae* in Peru (*11*), *L. wolffii* in Thailand (*14*), and *L. inadai* (*12*). Severe leptospirosis symptoms have been associated only with the intermediate cluster species *L. broomii* (*15*).

Febrile symptoms could be caused by many infectious agents (*17*), as evidenced by our finding that in Guayaquil and Portoviejo, 80% and 57% of the febrile population, respectively, did not show evidence of leptospirosis or dengue infection. In addition, environmental factors in these communities may facilitate exposure of inhabitants to multiple infectious agents; thus, febrile symptoms may be due to co-infections. We found evidence of concurrent dengue virus (IgM ELISA) and *Leptospira* spp. (PCR) infection in 27% of serum samples from Esmeraldas and 22% from Portoviejo. However, concomitant positive diagnostic outcomes for leptospirosis and dengue might be due to persistent presence of antibodies. Detection of IgM antibodies to dengue virus starts 4–5 days after the onset of symptoms and extends for up to 5 months after infection (*20*), whereas PCR for *Leptospira* spp. on blood samples mainly detects acute infection (*21*) and, as reported by others (*22*,*23*), is unsuitable for detecting asymptomatic renal colonization. Thus, while co-infection cannot be ruled out, it is conceivable that most, if not all, of these co-infected febrile patients had acute leptospirosis.

We also found high carriage rates of intermediate leptospires (*L. inadai* and *L. wolffii*) among domestic and peridomestic animals in Portoviejo in 2009 and 2013 (Figure; Table 3). This finding concurs with those in published reports showing intermediate leptospires carried by domestic and peridomestic animals (*11*,*13*,*24*). The meaning of the relative proportion of intermediate cluster strains observed in this animal study must be considered with caution as we cannot exclude the possibility of selection bias, given the fact that animals were not randomly sampled.

 We present molecular evidence of the presence of a similar intermediate *Leptospira* spp. (*L. wolffii*) among animal populations and humans in the same locality (Portoviejo). However, because the sampling was conducted at different times, we were unable to directly link *Leptospira* spp. carriage among animals and humans. This linkage is further complicated by a difference in prevalence rates of *L. wolffii* DNA among humans in 2012 and animals in 2013 (24% and 2.6%, respectively) (Figure). The difference in distribution of *Leptospira* spp. in humans and animals may be caused by human lifestyle, which can reduce direct or indirect exposure to the animals, or by different environmental survival capacities of pathogenic and intermediate *Leptospira* spp. (*25*). Nevertheless, we showed presence of the same intermediate DNA species of *Leptospira* in humans and animals, which is consistent with findings in other studies that suggest a link between human disease caused by intermediate leptospiral species (*L. licerasiae*) from rats and water sources in Peru (*3*,*11*).

The presence of leptospiral species in animals appears to be temporally dynamic. In Portoviejo, we observed that the dominant leptospiral species shifted from *L. inadai* in 2009 to *L. borgpetersenii* and *L. wolffii* in 2013 (Table 3). Temporal changes in leptospiral sequence types have been previously reported (*26*). It is possible that environmental conditions (e.g., humidity, intensity of rainy season, abundance of some animal species, chemical changes in natural water sources) may favor colonization of reservoir animals with a given type of leptospires. These temporal dynamics may explain the apparent sporadic nature of leptospirosis outbreaks; the circulation of pathogenic *Leptospira* spp. may cause typical and easily recognizable disease, whereas the circulation of intermediate species may cause a generally milder disease with a broad spectrum of symptoms, which makes the disease prone to misdiagnosis. These results highlight the need to conduct longitudinal surveys of leptospiral populations.

Differences in sanitary infrastructure may explain the higher prevalence of leptospiral infection in the rural community as compared with the more urban communities (Table 2). Communities in Esmeraldas tend to rely more on rivers for fresh water and transportation, increasing the probability for leptospiral exposure. Because both urban and rural communities co-exist with animals that carry leptospires, the difference in prevalence we observed likely reflects the efficiency of leptospiral dispersal by water. Unfortunately, we were unable to obtain information about water exposure or occupation of the febrile patients.

Our study also lacked clinical data for febrile patients, which prevented the investigation of presumptive leptospirosis–dengue co-infections or of the difference between infections with pathogenic or intermediate leptospiral species. Although these limitations prevent us from drawing stronger conclusions, our study clearly showed compelling evidence of the abundant presence of intermediate *Leptospira* spp. in humans and animals. This finding warrants further investigation of the effect of these species on the disease burden observed in veterinary and human public health.

Other limitations in our study were the low number of DNA sequences obtained in 2009 from animals in Portoviejo and the lack of leptospiral isolates belonging to the intermediate cluster. A year later, we attempted without success to amplify leptospiral sequences from positive samples. Also, despite many attempts, we failed to isolate intermediate *Leptospira* spp. from febrile humans and domestic animals, although we isolated *L. santarosai* (pathogenic cluster) from a dog urine sample collected in Portoviejo in 2009 (data not shown). It is possible that intermediate species circulate at lower numbers than pathogenic counterparts or that some of these species may be more fastidious than pathogenic species.

Intermediate leptospires are rarely detected in humans, probably because many PCR protocols amplify genes that are present only in pathogenic species (*21*). Genetic characterization of *Leptospira* spp. makes it possible to understand disease transmission patterns and to obtain new insights by reinterpreting serologic and clinical epidemiologic data within a genetic context. Correct identification of the etiologic agent is critical for disease management in regions where dengue, malaria, leptospirosis, and, more recently, chikungunya are present (*27*,*28*). Our finding of a high number of false-positive reactions reveals the risks of using the 16S PCR (without amplicon sequencing) for diagnosis of leptospirosis.
